# Mitochondrial Dysfunction and Oxidative Stress in Aging and Disease

**DOI:** 10.3390/biomedicines10112872

**Published:** 2022-11-09

**Authors:** Yi-Ling Tsang, Chiu-Li Kao, Shu-Chuan Amy Lin, Chia-Jung Li

**Affiliations:** 1 Institute of Physiological Chemistry and Pathobiochemistry and Cells in Motion Interfaculty Centre (CiMIC), University of Münster, 48149 Münster, Germany; 2 Department of Nursing, Tzu Hui Institute of Technology, Pingtung County 926, Taiwan; 3 Department of Nursing, National Yang Ming Chiao Tung University Hospital, Yilan 260, Taiwan; 4 Nursing School, National Yang Ming Chiao Tung University, Taipei 112, Taiwan; 5 Department of Obstetrics and Gynecology, Kaohsiung Veterans General Hospital, Kaohsiung 813, Taiwan; 6 Institute of BioPharmaceutical Sciences, National Sun Yat-sen University, Kaohsiung 804, Taiwan

Mitochondria are considered to have a significant influence on aging due to their critical role in the regulation of bioenergetics, oxidative stress, and cell death. Mitochondrial oxidative stress, commonly associated with age-related pathologies (neurodegenerative syndromes, cardiovascular diseases, endocrine pathologies, diabetes, and cancer), can damage mitochondrial DNA, proteins, and lipids. The increased ROS presence can also induce chronic inflammation, which often characterizes age-related diseases and autoimmune pathologies. Therefore, it is important to understand new and available molecular mechanisms, their effects on the antioxidant process, and in particular, how they protect cells and organs from the harmful effects of free radicals to treat disease.

Mitochondria quality control is important for modulating the progression of diseases such as Parkinson’s disease (PD). Specific gene expressions such as PINK1 and PRKN are major genes that regulate mitochondrial quality control. Heng-Chung Kung et al. highlight several PD-associated genes that also mediate mitochondria function [1]. This review reinforces the importance of failure control of mitochondria function and oxidative stress contributing to neurodegenerative disease. The authors propose a set of mitochondrial oxidative stress mechanisms and discuss their relationship with Parkinson’s disease. Notably, mitochondrial oxidative stress is important in neurodegenerative diseases, and antioxidant compounds and polyphenols are becoming a potential therapy for these diseases. Furthermore, the authors demonstrate the neuroprotective properties of polyphenols and their ability to reduce oxidative stress, with an in-depth discussion of resveratrol. Ultimately, they also pointed out that resveratrol exhibits biphasic concentration-dependent effects, and there is an uncertainty in the translation of pre-clinical study to clinical trials; hence, there is a limited number of clinical studies on resveratrol.

Salubrinal is an inhibitor of eIF2α dephosphorylation and an inducer of ISR. Mei-Chun Chen et al. investigated the intervention of salubrinal in cancer cell death by activating the integrated stress response (ISR) and mitochondrial oxidative stress [2]. They found that salubrinal did not suppress cancer cell growth in a glucose-containing condition. However, when salubrinal induces ISR under glucose deprivation, it also induces cell death and increases levels of mitochondrial reactive oxygen species (ROS). In the previous study, the upregulation of protein synthesis was found under conditions of mitochondrial stress condition. The authors detected an upregulation of xCT and oxidative-stress-mediated cell death during glucose deprivation after the salubrinal treatment. These findings elucidate the role of salubrinal and mitochondrial oxidative stress in cancer cell death under glucose deprivation. This also indicates that mtROS is an important mediator for cancer cells to adapt to nutrient-limited conditions. From a clinical perspective, combining the treatment of salubrinal with a chemical that diminishes glucose metabolism is a novel therapeutic approach to cancer.

The previous study found that mitochondria function is decreased in diabetic kidney. Excess glucose induces mitochondrial dysfunction and leads to cell damage and inflammation. Carla Iacobini et al. hypothesize that a high level of glucose induces hypoxia inducible factor (HIF)-1α activity, initiating glucotoxicity [3]. In the in vitro study, HIF-1α was increased after the incubation of high glucose. They further used methylglyoxal (MGO), a non-hypoxic inducer for HIF1-α, to reproduce the effects of high-glucose induction. This indicates that MGO is able to switch oxidative metabolism to glycolysis. The mechanism of MGO in the pathway of HIF1-α causes prolyl 4-hydroxylase domain 2 (PHD2) to interact with HIF1-α, leading to HIF1-α degradation and diminishing enzyme activity. Together, this article provides a new treatment strategy in diabetes by targeting HIF-1α or PHD2. However, a further preclinical study is still needed to thoroughly confirm this mechanism.

Mitochondrial dynamics are mediated by the process of fission and fusion, further controlling cellular homeostasis. Giovanni Fajardo et al. cover the mechanisms underlying mitochondria quality control and links to pathologies. Maintaining mitochondria homeostasis needs both clearance and biogenesis. In order to maintain healthy mitochondria, mitophagy is an important protective action that removes dysfunctional mitochondria. In this context, the authors summarized the alterations of mitochondrial quality control and its prevalent cardiovascular disease model [4]. Both mitochondria fission and fusion can cause cardiac dysfunction, thus indicating that the new therapeutic strategy is to maintain the balancing of mitochondrial remodeling in heart. The inhibition of mitochondrial fission by small molecules attenuates cardiac injury and improves bioenergetics in ischemic models. On the basis of fission inhibition, the induction of mitochondrial fusion improves ischemia–reperfusion treatment by reducing ROS production, resulting in improved mitochondrial function. As well as modulating fission and fusion, Spermidine treatment in mice showed a stimulation of protective autophagy and mitophagy in cardiomyocytes; However, these protective effects are subordinated by several mechanisms. Giovanni Fajardo et al. thoroughly discuss mitochondria dynamics in pharmacologic modulation and the mechanism of balance in maintaining mitochondrial homeostasis. Unanswered questions remain about the off-target effects of these interventions. Nevertheless, new research in this field could provide new pharmacological alternatives for manipulating the mitochondria function.

Intracellular oxidative stress is not only from the mitochondria, but also from the endoplasmic reticulum, which is a source of ROS. Endoplasmic reticulum (ER) stress is also a major cause of renal cell injury during the progression of acute kidney injury. Moreover, the excess reactive oxygen species (ROS) that cause oxidative stress may increase the susceptibility of the aging kidney to prolonged ER stress-induced acute injury. Chih-Hung Lin et al. found that crysophanol prevented H/R-induced cell death via apoptosis by increasing the expression of Bax and p-JNK but decreasing the expression of Bcl-2, as well as prolonging ER stress by increasing the expressions of CHOP and p-IRE1 [5]. The authors found that hypoxia–reoxygenation (H/R) triggered ferroptosis through the accumulation of lipid ROS and the downregulation of anti-ferroptotic molecules GPX4 and SLC7A11, which induced the death of HK-2 cells. On the other hand, chrysophanol showed a potential anti-ferroptotic effect on HK-2 cells under H/R conditions by upregulating GPX4 and SCL7A11 and reducing lipid ROS accumulation. Therefore, the present study may demonstrate a protective effect against renal injury through the regulation of apoptosis, endoplasmic reticulum stress and ferroptosis by chrysophanol.

Mitochondria function is linked to aging due to mitochondria DNA mutation and decreased enzyme activity. SIRT3 activity is related to human longevity and is located in the mitochondria matrix. Research shows that SIRT3 regulates antioxidation, amino acid metabolism, and mitochondria permeability proteins. Ciprian N. Silaghi et al. discussed SIRT3 in aging biology and the intervention of age-related diseases by activating SIRT3 [6]. The overexpression of SIRT3 inhibits HIF1-α upregulation, further decreases Ve-cadherin, and normalizes endothelial cell permeability. The authors listed the intervention of SIRT3 in cardiac hypertrophy models and summarized its effect. In this study, SIRT3 reduced the extent of myocardial atrophy and protected cardiac myocytes from damage. In general, SIRT3 is an important enzyme that has antioxidant ability, maintaining mitochondria function and protecting against cardiac hypotrophy. Moreover, SIRT3 is abundant in the nervous system, which is a negative regulator in age-related neurodegenerative diseases. Several interventions of SIRT3 signaling show that it improves mitochondria function and provides a protective effect in neurodegenerative diseases.

Glutamate dehydrogenase (GDH) is an oxidoreductase and is treated as a stress-response enzyme. There are several cellular processes involved in GDH, including ATP regulation and the interconnection between nitrogen and carbon metabolism. However, the function of GDH in metabolic pathways in cancer progression is still unclear. Michela Marsico et al. elucidate the mechanism of GDH1, one of the isoforms of GDH, in regulating hepatocellular carcinoma (HCC) [7]. They primarily found that GDH1 is more abundant in diseased liver compared to normal liver. The proliferation of cells decreased when GLUD1 gene was silenced. However, there is no cytotoxic effect on human hepatocytes after GLUD1 gene silencing. This indicates that GLUD1 gene reduces proliferation by apoptosis. The authors also prove that apoptosis is related to BCL2 downregulation through mitochondrial changes. Overall, the intrinsic pathway of apoptosis is driven by GLUD1 gene silencing. The authors provide a new strategy that could impair the metabolic reprogramming of HCC by inhibiting GLUD1.

Ferroptosis is a type of cell death regulated by an iron-dependent signaling pathway. Distinct from other types of cell death, ferroptosis does not present cellular swelling nor shrinkage in the same way as necroptosis and proptosis. However, it demonstrates a disorganization of mitochondria, such as mitochondria shrinkage, the vanishing of mitochondria cristae, and the rupturing of the outer mitochondria membrane. The dysregulation of iron metabolism is related to cellular damage and oxidative stress, which is especially found in the disease progression of PD. Furthermore, in PD animal models, it was found that ferroptosis is a cell death pathway for dopaminergic neurons. The review by Chih-Jan Ko et al. provide a detailed summary of the ferroptosis pathways linked with neuroinflammation and PD and summarize the recent clinical trials targeting ferroptosis [8]. As an emergency pathway in PD, further investigation of ferroptosis-related biomarkers is important in this field. Although several biomarkers from CSF and blood are discovered in PD progression, ferroptosis-specific pharmacodynamic biomarkers and translational strategies for treatment remain unknown.

Antioxidant agent is a negative modulator of ROS. Idebenone is an antioxidant that could maintain ATP production and disturb lipid peroxidation in brain mitochondria. Retinal pigment epithelium (RPE) is an epithelial cell with a pigment that absorbs light and provides a barrier between the choroid and retina. Interestingly, there are abundant mitochondria in RPE, providing better resistance to oxidative stress. Maria Elisabetta Clementi et al. investigated the correlation of Idebenone in support of mitochondria homeostasis and its cytoprotective effect on oxidative stress in RPE [9]. Initially, they found that the Nrf2 signaling pathway, which responds to oxidative stress, was activated after Idebenone stimulation. Moreover, the addition of Idebenone restored the mitochondrial membrane potential under H_2_O_2_-induced mitochondrial depolarization. The intrinsic apoptotic pathway was also analyzed, and Idebenone significantly inhibited the release of cytochrome-c and Caspase-3 activity. Overall, Idebenone maintained the activity of respiratory chain complexes and antagonized the Caspase-3 activation in response to oxidative damage, suggesting its positive regulation with mitochondrial dysfunction.

Mitochondria dysfunction leads not only to neurodegeneration disease but pulmonary emphysema. Pulmonary emphysema is a chronic lung condition that appears in overinflated air sacs and destroys alveoli. Alveolar type II (ATII) cells contain abundant mitochondria. Previous research found that OXPHOS complexes are encoded by mitochondria genome and its complex I modulate energy metabolism. Loukmane Karim et al. isolated ATII cells from control non-smokers and smokers, and emphysema patients. [10] They found ND1 was increased and UQCRC2 was decreased in lung tissue from patients with emphysema compared to non-smokers. To understand the pathway that triggers apoptosis in emphysema patients, they analyzed the active caspase 9 levels in ATII cells and found its higher expression in emphysema patients compared to controls. This showed that mitophagy may trigger apoptosis. They further detected ND1, UQCRC2, and COX4 and analyzed the mitochondria function. All of these genes are decreased in emphysema patients compared to smokers. The increased nuclear NDUFS1 and SDHB levels and the decreased mitochondrial ND1 protein indicate the impairment of the nuclear/mitochondrial stoichiometry in ATII cells in emphysema. Mitoribosome structural protein MRPL48 is low in ATII cells in emphysema. They also detected a decreae in 16S rRNA expression and an increase in inc2S rRNA levels. Moreover, miR4485-3p is analyzed in emphysema and shows a negative feedback loop with 16S rRNA. This research revealed a molecular mechanism of mitoribosome dysfunction in ATII cells in emphysema.

Although several studies elucidated the critical function of mitoribosome, its diversity in different cells and diseases continues to emerge. Much remains to be researched in the field of mitochondrial biology regarding quality control, aging, and homeostasis. In this Special Issue, we aim to provide a perspective on the current trends of this complex topic and unanswered questions driving mitochondrial research.
